# A Practical Treatise on Phthisis Pulmonalis

**Published:** 1861-09

**Authors:** 


ART. III.—*A Practical Treatise on Phthisis Pulmonalis, embracing its Pathology, Causes, Symptoms and Treatment.* By L. M. LAWSON, M.D., Prof. of Clinical Medicine in the University of Louisiana; Visiting Physician to the New Orleans Charity Hospital; formerly Professor of the Theory and Practice of Medicine in the Medical College of Ohio. 1 vol., 8vo., pp. 560. Cincinnati: Rickey, Mallory & Co.

A NEW work on phthisis pulmonalis, at the present time, may seem to be uncalled for. Already a large number of able treatises on the subject are before the profession; indeed, so much has been written, and so little ascertained as to the true nature of its cause and its successful treatment, that it is difficult for any new candidate for public favor to obtain a patient and unbiased hearing. In the work before us, Dr. Lawson, although he presents some new and interesting views, and has done much toward placing the whole subject on a more scientific basis, does not claim to have demonstrated any new fact; nor does he suggest any novelty as to its treatment, which, of itself, is calculated to inspire us with new hope or to give a distinctive character to his book; and yet we think no one can rise from its perusal without being thoroughly convinced that we needed just such a book. In the general plan of the work; in the thorough, careful, and logical analysis of all known facts; in the fullness, clearness, and discriminating character of the diagnostic principles; in the systematic description of the precursory stage of the disease; in its valuable statistical tables; and in the judicious application of the treatment as applied to the different stages of the disease, it is doubtless greatly in advance of any work of the kind that has ever been offered to the profession.

The first thing that favorably impresses the reader, in opening Dr. Lawson's book, is its admirable arrangement. It is divided into four general parts, and these are subdivided into chapters and sections, a full and complete index being appended to each, so that the reader can, at a glance, see the general scope of the work, and turn to any subject which he may desire.

Part first embraces the Pathology of Phthisis. This is divided into chapters, including a full discussion of the tuberculous constitution, of the precursory stage of phthisis, of tuberculous deposits, the condition of the blood, the state of the secretions, the deposit of tubercle, changes which occur in tubercular deposits, changes consequent upon softening and elimination of tubercle, distribution of tubercle, secondary and intercurrent lesions, tertiary lesions, forms or varieties of phthisis, and the general nature of phthisis.

Part second embraces the Etiology of Phthisis, including the discussion, in separate chapters, of the general predisposition to phthisis, of the causes which may induce phthisis independent of hereditary predisposition, such as climate, temperature, humidity, altitude, atmospheric impurities, temperaments, age, sex, occupation, ingesta, &c.; and of pathological inducing causes.

Part third embraces the Semeiology of Phthisis.

Part fourth the Therapeutics of Phthisis.

In the chapter on the general nature of phthisis the author examines, at length, all the more popular and captivating theories of tuberculosis. The subject of impaired primary digestion, imperfect development of chyle, morbid condition of lymph, defective respiration, morbid states of the blood, specific poison, retrograde morphology, inflammation, etc., are all clearly presented, and the arguments for and against fairly and candidly considered. Dr. Lawson devotes a large portion of this chapter to the refutation of the popularly received view which refers phthisis to errors of *primary digestion.* From the *gastric* origin of the disease he entirely dissents; and after stating his objection to this, and many of the commonly received opinions—indeed all of them—he arrives at the conclusion that there is but one remaining source to which we can look, namely, the *metamorphosis of the tissues.*

"Taking all these facts into consideration," says Dr. Lawson, "I am led to conclude that tubercle is almost of necessity the product of the metamorphosis of the tissues. It is here, and here alone, that we find those important changes taking place which result in new combinations, chemical and organic, and which, passing into the blood, lead to local disease. This may be termed a *diathesis* when it is a result of a peculiar constitutional conformation, which, by its own natural tendency, eventuates in a specific form of disease."

Mere blood-changes are not regarded as sufficient to constitute either the tuberculous condition or the local deposits; and analysis has failed to point out the elements of tuberculous matter in the blood. But on the supposition that the elements of tubercle are formed in the metamorphosis of the tissues, and that these elements, seeking elimination through particular tissues, become deposited or arrested, with all the attending phenomena of tubercle, all difficulties, in the estimation of our author, are removed, and "the subject assumes a clearer and more philosophical aspect." Corroborative evidence of the correctness of these views is also deduced from the irritation of the lymphatic system, which is often present in the tuberculous diathesis. The identity or close similarity of scrofula and phthisis also receives a passing notice.

"In examples of *tuberculosis*, the morbid element enters the venous radicals, passes into the general circulation, and reaches the lungs and other tissues; in *scrofulosis*, the irritant enters the lymphatics, affects the glands, and gives rise to all the signs of that disease. To what extent these two conditions may act reciprocally, or how far the predominance of one may suspend or modify the other, are important but undecided questions; but there are some facts which indicate that a decided impression produced on one system affords some relief to the other; and thus scrofula often exists without turberculosis, and *vice versa*. It is not improbable, indeed, that if the morbid element takes the course of the external lymphatics, the internal structures may escape, and thus these vessels, acting as a diverticulum to the lungs, actually afford a protection to those organs."

No explanation is attempted of the law by which, in one instance, the morbid element enters the lymphatics and develops external scrofula, while, in another, it passes into the general circulation, and finally involves the lungs. Nor does the want of explanation alone furnish a valid argument against the views here presented; for as strong, or even stronger objections may be offered to any other theory of tuberculosis.

Finally, after an elaborate discussion of the pathology of phthisis, in which all the prominent and popular theories are carefully reviewed, Dr. Lawson seems to think the following conclusions are fairly deducible from the facts which he has collected:—

"1. The tuberculous element originates in the metamorphosis of the tissues.

"2. It seeks elimination through the lungs, and may continue to pass, in certain quantities, for an indefinite period, without inducing local deposits.

"3. When the morbid element reaches a certain degree of concentration, or when, by long-continued action, it produces a morbid effect on the lungs, local deposits take place.

"4. The first deposit is the elementary morbid substance known as the amorphous stroma; this is followed by the development of molecular granules and peculiar cells, which constitute tubercle.

"5. After the existence of solid tubercle for a given period, it softens, and the debris seeks elimination through the bronchial tubes.

"6. The morbid action extends to the adjacent tissues, causes inflammation, softening, and disintegration, too often resulting in fatal disorganization.

"7. The perfect uniformity of tubercle throughout the body, in whatever tissue or organ deposited, exhibits strong evidence of the specific character of the disease, and that it could not originate from the ordinary derangements of nutrition.

"8. The chemical and histological character of tubercle favor the opinion that the whole process is specific in origin and development."

In the second general division of the work, embracing the ETIOLOGY of PHTHISIS, there is an exceedingly interesting chapter on what the author terms "the geography of phthisis," by which he means to indicate the natural prevalence of the disease among the permanent inhabitants of a given district of country. The statistical tables, carefully elaborated from the United States Census, showing the relative prevalence of phthisis in the different parts of the United States, comprise the fullest and most complete collection of facts on the subject that has ever been presented to the profession of this country; and, if the work had no other merits, this alone should entitle it to a place in every American medical library.

The opinion which has long prevailed that climate exercises an influence over phthisis, is amply confirmed by Dr. Lawson. Two facts are regarded as established so far as temperature is concerned, namely, that the extremes of heat and cold are conditions unfavorable to its production; hence, *cæteris paribus*, the arctic and tropical regions are most exempt from phthisis; while, on the contrary, the disease prevails to the greatest extent in the so-called temperate latitudes. In Lapland, consumption is said to be a rare affection, and in Great Britain and Ireland it is much more common than in Russia. Some very interesting facts are presented as to the prevalence of phthisis in the arctic regions, in Europe, Asia, India, Africa, and the West Indies. But to the American reader, the most interesting feature of the work is the extensive collation of facts as to the prevalence of consumption in the different regions of the United States. The statistics are mainly drawn from the United States Census Reports, Army Reports, Drake's Diseases of the Interior Valley, Blodget's Climatology, and reports of local authorities; and the profession are indebted to Dr. Lawson for the finest collection of statistical information on this subject that has ever been presented in this country.

The main fact developed by these statistics is, that phthisis, in the United States, progressively decreases from North to South. While it is freely admitted that the facts upon which this opinion is based cannot be regarded as more than approximative, they nevertheless furnish sufficient data, in the author's judgment, to warrant the general conclusion at which he arrives.

"I am fully persuaded that consumption originates far less commonly in the Southern than in the more Northern regions, and that it gradually but perceptibly diminishes from Maine to Florida; and, without claiming absolute accuracy, I believe the tables compiled from the United States Census are very near the truth, and will be found sufficient guides for practical purposes. Thus, the mortality from phthisis in the eastern division may be set down at one in three hundred and twenty-eight; in the middle division, one in five hundred and nineteen; in the western division, one in eight hundred and eighty-two; and in the southern division, one in twelve hundred and eighty-seven. This shows the mortality from phthisis to be three times greater in the northern than in the southern division."

And he adds:—

"Another fact has been brought to light by these statistics which, probably, had not been generally anticipated, namely, that the mortality from inflammatory diseases of the chest, mainly pneumonia, is much greater at the South than at the North. Thus, we find the mortality from diseases of the respiratory organs in the eastern division, one in fourteen hundred and fourteen; in the middle division, one in two thousand four hundred and twenty-eight; in the western division, one in twelve hundred and forty-eight; and in the southern division, one in eight hundred and sixty-five."

Some ingenious speculations are offered in explanation of the effects of heat and cold in their relation to phthisis. In the cold atmosphere of the arctic regions a larger proportion of oxygen is consumed. In hot climates, on the contrary, the quantity of oxygen is relatively diminished, the blood is not so thoroughly decarbonized, the tissues break up slowly, and tubercular matter is not formed. In high northern latitudes, while a larger quantity of oxygen is consumed, the diminished temperature necessarily retards the organic movements. In addition to this, the inhabitants during the cold season take little exercise and live on highly carbonaceous articles of food; hence the diminished oxidation and consequent diminished metamorphosis of the tissues.

"It seems a fair inference, that the influences of heat and cold in protecting the system against the tubercular diathesis operate by virtue of the diminished transformations of the tissues, depending, in one case, on the protective agency of carbon, and in the other, on the depressing effects of cold. In either case the metamorphosis of the tissues becomes comparatively slow, and the tuberculous material scantily supplied."

Another distinctive character of the work before us consists in the systematic description of the *precursory stage* of phthisis. Dr. Lawson lays great stress upon this point. That the disease may be detected, even in its forming stage, he thinks can be fully demonstrated. In this stage he relies, mainly, on the general or rational symptoms: it is only in the more advanced stages that we have the more marked and prominent symptoms, and then, as justly remarked by the author, we accomplish little more than discover an incurable affection.

The precursory stage is described as "intermediate between the mere diathesis on the one hand, and the development of solid tubercles in the lungs on the other." In this stage, according to Dr. Lawson, the vital powers become impaired, the general strength diminishes, there is gradual loss of weight, irregular chills, followed by slight febricula, debility of the heart, arteries, and capillaries, irregular and morbid excitability, and a peculiar morbid condition of the mucous membrane and glandular structures of the fauces and larynx, which very constantly precedes the pulmonary affection. Much importance is attached to this latter symptom, although it is not regarded in the light of a cause, but as a mere local expression of a constitutional predisposition.

Numerous cases have come under the author's observation in which copious hemorrhage has occurred during the precursory tubercular stage.

Can this precursory stage be detected by *physical signs?* Dr. Lawson answers emphatically in the affirmative. In the first place, the condition of the chest appears to be one of debility of the moving powers of the parietes, and, as a consequence, there is limited expansion, *extending equally to both sides.* Expansion is comparatively small, and the movement proportionally less at the apex than the base; but full inspiration restores, almost perfectly, the harmony of the movement. After tubercles have been deposited the expansion of the apex cannot be restored, even by forcible inspiration; nor is the action that of a gradual swelling from below upward, as in the precursory stage.

In consequence of diminished mobility of the thorax, the sound elicited by percussion is less clear than that of the physiological type, and is equal on the two sides; but forcible inspiration will either absolutely remove or greatly reduce this dullness.

Auscultation develops weak respiratory murmur and jerking inspiration.

"Finally," says Dr. Lawson, "my observation has taught me to believe that the precursory stage of phthisis may be readily recognized; that it has well-marked constitutional symptoms and clearly-defined physical signs. The constitutional symptoms embrace loss of weight, irregular chills, febricula, variable appetite; while the local signs include faucial and laryngeal derangements, cough, hæmoptysis, diminished expansion of the chest, weak and jerking respiratory murmur. These symptoms, occurring in a constitution predisposed to phthisis, can admit of little doubt or misconstruction."

Space will not allow us to present an extended synopsis of the *Therapeutics of Phthisis*, as given by the author, which, after all, is the best part of the book. We have no work on the subject, which, in our judgment, is so full in its details of treatment, or in which the principles of treatment are applied to the different stages of the disease with such just discrimination. And especially is this true of the treatment of the *precursory* stage.

The chapter on *Change of Climate*, in its relations to the different stages of consumption, is a most valuable contribution to our literature on that subject. It may be safely affirmed that we have no other work which presents this subject in so rational, clear, and philosophical light.

The author closes the subject of treatment with the following paragraph:—

"But unfortunately a large, perhaps the larger number, at least in this country, do not seek reliable aid until they have passed the period of cure, or even of palliation. Our country abounds with designing charlatans, who falsely proclaim their ability to cure consumption with certainty and facility; and the press teems with alluring advertisements and inspiring certificates, proclaiming the discovery of specifics, before whose magic power disease recedes, as the night before the rising sun. And pale victims crowd these halls of false promises and heartless deceptions, eagerly grasping the gilded bubbles; but, alas! the dream is not realized, and the deluded victim finds relief in his narrow house, while the heartless mountebank goes on dispensing false promises, and reaping a golden harvest."

We have aimed, in this hurried review, to present, as compactly as possible, some of the peculiarities of Dr. Lawson's work rather than to give an extended review. It is a work of substantial merit, and reflects great credit on American authorship. In its admirable arrangement, clearness of style, and close and logical analysis of all known facts pertaining to the subject of which it treats, it is a model book, and should be in every library.

